# Work-related interventions for preventing back pain—protocol for a systematic review and network meta-analysis

**DOI:** 10.1186/s13643-021-01768-5

**Published:** 2021-08-30

**Authors:** Angelika Eisele-Metzger, Daria S. Schoser, Kathrin Grummich, Guido Schwarzer, Lukas Schwingshackl, Bianca Biallas, Christiane Wilke, Joerg J. Meerpohl, Cordula Braun

**Affiliations:** 1 Institute for Evidence in Medicine, Medical Center - University of Freiburg, Faculty of Medicine, University of Freiburg, Freiburg, Germany; 2 Cochrane Germany, Cochrane Germany Foundation, Freiburg, Germany; 3 Institute of Movement Therapy and Movement-Oriented Prevention and Rehabilitation, German Sport University Cologne, Cologne, Germany; 4 Institute of Medical Biometry and Statistics, Faculty of Medicine and Medical Center, University of Freiburg, Freiburg, Germany

**Keywords:** Back pain, Musculoskeletal pain, Primary prevention, Occupational health, Workplace, Network meta-analysis, Systematic review

## Abstract

**Background:**

Back pain is a widespread health problem that accounts for substantial disability and high costs. The workplace is considered to critically affect the occurrence and persistence of back pain and therefore offers an important opportunity for preventive interventions. Various work-related intervention strategies including both single- and multicomponent interventions have been developed and evaluated so far. To determine their effectiveness, a method of analysis is needed that particularly meets the challenges of the multidimensionality and diversity of these interventions. This planned systematic review and network meta-analysis aims to compare the effects of different work-related interventions for preventing non-specific back pain in people within a formal employment-related context.

**Methods:**

We will search the following databases: CENTRAL, MEDLINE, Web of Science, CINAHL, PsycINFO, PEDro, SPORTDiscus, and Academic Search Premier from their inception onwards, as well as additional sources. Randomized controlled trials (RCTs) and cluster-RCTs will be considered if they (1) include people within a formal employment-related context, (2) include people without back pain or mixed samples (i.e., people with and without back pain), (3) compare one or more work-related preventive intervention(s) to a control condition, and (4) assess non-specific back pain (incidence or/and pain intensity), ability to work (numbers of participants or/and numbers of days absent from work), intervention-related adverse events or/and self-reported satisfaction with the intervention. Random-effects pairwise meta-analyses and frequentist network meta-analyses will be conducted where appropriate. We will calculate summary effect sizes for each comparison of interventions and rank interventions according to their *P* scores. If feasible, we will conduct additional component network meta-analyses. We plan to conduct subgroup analyses for job exposure, intervention duration, baseline back pain, different localizations of back pain, and gender. Risk of bias will be assessed using RoB 2 and the certainty of the evidence will be rated using the GRADE approach.

**Discussion:**

This systematic review aims to identify work-related intervention strategies as well as components within work-related interventions that are effective for preventing back pain. We expect the results to provide guidance for selecting the most promising interventions and foster the purposeful use of resources. Additionally, they may inform the development and implementation of work-related interventions as well as the design of future research in this field.

**Trial registration:**

PROSPERO CRD42021232469

**Supplementary Information:**

The online version contains supplementary material available at 10.1186/s13643-021-01768-5.

## Background

Low back pain and neck pain cause high numbers of years lived with disability worldwide and among most age groups [1, 2]. In 2017, over half a billion people worldwide suffered from low back pain [3]. The prevalence of back pain and related disability has substantially increased over the past years and is likely to increase further as the population ages [1]. Back pain is commonly recurrent and true remission in terms of lifelong full recovery is rare in the majority of cases [4]. The economic burden of back pain is substantial, with particularly indirect costs due to lost work productivity contributing to the overall costs [5]. In Germany, musculoskeletal disorders such as back pain cause the highest number of days of work absence among all diseases and are the second most common reason for health-related early retirement [6].

Different work-related factors have been identified as risk factors for the occurrence and/or persistence of back pain, including physical factors such as manual handling, whole-body vibration, awkward posture, bending and twisting of the trunk, and prolonged sitting [7]. Beyond these, also psychosocial factors such as low social support, job dissatisfaction, or high job demands need to be considered [7]. Consequently, work-related programs to prevent back pain have gained increased attention over the past years [7, 8]. In particular, exercise programs conducted in workplace settings have been shown to be beneficial in preventing back pain [9, 10]. Other interventions, such as ergonomic workplace interventions or educational programs have not yet demonstrated substantial effects [9, 11]. In a review of back pain prevention and management strategies in the workplace, Schaafsma et al. [7] emphasized the common multifactorial origin of back pain and the variety of possible influencing factors. They proposed that workplace interventions should address a combination of physical and psychosocial risk factors and use a more comprehensive approach in order to generate significant effects on the incidence of back pain [7]. Indeed, previous systematic reviews [12, 13] have found work-related interventions that include multiple intervention components to be effective in preventing back pain. However, evaluating multicomponent interventions as well as identifying the individual components that are decisive for their effectiveness is often challenging [14]. Work-related multicomponent interventions for preventing back pain commonly include exercise as an intervention component [12, 13]. The added value of any further components included in these interventions is yet unclear and has not been assessed in the available systematic reviews [12, 13]. An analytical approach that accounts for the multidimensionality of interventions as well as the variety of intervention strategies is needed when investigating the effects of work-related interventions for the prevention of back pain.

Network meta-analysis (NMA) is an extension of pairwise meta-analysis that is increasingly used in health research [15, 16]. It enables the simultaneous comparison of multiple intervention strategies and their ranking according to their relative effects [16]. Moreover, NMA allows to determine the relative effects of one intervention compared to another in cases where direct comparisons from original studies are missing using indirect comparisons within the network of original studies [16]. As an additional option, the method of component network meta-analysis (CNMA) offers the possibility to break down multicomponent interventions into their individual components in order to test their relative effects within the network of studies [17]. CNMA therefore enables the identification of effective intervention components within those interventions.

Prior to developing this systematic review, we reviewed available recent systematic reviews in the field of work-related interventions for back pain [9–11, 18–20]. This showed that these reviews largely either focus on specific interventions (e.g., exercise [10], breaks [20]) or populations (e.g., office workers [20]), do not specifically focus on interventions delivered within workplace settings [11, 18, 19], or do not include meta-analyses or (component) NMA [9]. To our knowledge, work-related interventions for preventing back pain have not been summarized in a systematic review with NMA so far.

This planned systematic review and NMA aims to investigate the effects of different work-related interventions for preventing non-specific back pain in people within a formal employment-related context. The specific aims are (1) to determine the effects of different work-related interventions on the incidence of non-specific back pain and on the ability to work as well as potential intervention-related adverse events and participants’ satisfaction with the interventions, (2) to combine direct and indirect evidence and rank different intervention strategies based on their effects using NMA, (3) to rate the certainty of evidence for the investigated outcomes, and (4) to explore potential differences in the effects of interventions depending on participants’ job exposure.

## Methods

This planned systematic review has a priori been registered with PROSPERO (CRD42021232469). This protocol is being reported in accordance with the reporting guidance provided in the Preferred Reporting Items for Systematic Reviews and Meta-Analysis Protocols (PRISMA-P) statement [21] (see checklist in Additional file 1). In reporting the methods and results in the final report, we will follow the PRISMA 2020 statement [22], and the extension for reporting network meta-analysis of health care interventions [23]. This protocol has been developed following Cochrane guidance for preparing a protocol for a systematic review with multiple interventions [24]. Any amendments to this protocol will be stated in the report of this systematic review.

### Eligibility criteria

We will include randomized controlled trials (RCTs) and cluster-RCTs fulfilling the following criteria.

#### Types of participants

We will include studies investigating populations of

- People within a formal employment-related context. We expect eligible studies to include participants aged 15 or older (reflecting the working population). However, in recognition of the known variability across settings and countries, we will not apply any explicit age limits. No restrictions regarding type of work will be made; however, military settings will be excluded due to their special work requirements and the anticipated difficulty of comparing them with other occupational groups.
- People without back pain at baseline. In recognition of the widespread prevalence and recurrent nature of back pain in the working population [4], we anticipate the inclusion of mixed samples (i.e., participants with and without back pain) in many otherwise eligible studies. We will therefore also consider studies including a proportion of participants with back pain, as long as all participants were able to work at baseline, or, where this is unclear or not reported, the back pain was, per definition of the study authors, of mild severity. We will exclude studies specifically designed to investigate people with back pain, i.e., studies in which all participants initially had back pain and/or were unable to work.

#### Types of interventions

We will include studies investigating work-related interventions aimed at preventing back pain and specifically designed for and delivered within a workplace setting. We plan to include the following types of interventions (categorization is based on the findings of two previous systematic reviews [9, 18]).

- Exercise programs (e.g., including strengthening exercises, endurance training, or flexibility training) [9, 18]
- Education (e.g., back schools, ergonomic training, advice on behavior change, or provision of information material) [9, 18]
- Ergonomic aids and adaptations (e.g., workplace adjustments, lifting aids, or scheduled breaks [20]) [9, 18]
- Orthoses, bandages, shoe insoles, or similar aids worn on the body [9, 18]
- Stress management interventions [9]
- Multicomponent interventions [9] including two or more of the interventions mentioned above (e.g., exercise program plus education).

Eligible comparators include no intervention/usual care or any of the interventions mentioned above. We plan to include these interventions in the NMA clustered according to the categories listed above. The interventions may be supervised or unsupervised and may be delivered individually or in groups. We will also include studies comparing different modes (e.g., different durations, frequencies, or intensities) of an intervention. Whether different modes can be considered separately for the NMA will be decided based on the available studies [24]. There may be other interventions relevant to our research question of which we are not aware at the time of writing this protocol [24]. We therefore plan to include relevant additional interventions “post hoc” in our NMA if we consider them to be comparable with the other pre-specified interventions [24].

#### Types of outcome measures

The primary outcomes of interest for our review are the following:

1. Back pain:
  1.1 Numbers of participants with at least one new episode of non-specific back pain.
  1.2 Intensity of non-specific back pain, measured with, e.g., a numeric or visual analog scale (NRS or VAS).
2. Ability to work:
  2.1 Numbers of participants put on sick-leave due to their back pain and/or due to any cause.
  2.2 Numbers of days with work absenteeism due to back pain and/or due to any cause.

We define non-specific back pain as pain in any part of the back, e.g., low back or neck pain, without a known specific pathology such as trauma, inflammatory conditions, or neoplastic causes [25]. The pain may extend from the back to other parts of the body, e.g., from the neck to the shoulder.

The secondary outcomes of interest for this review are the following:

1. Intervention-related adverse events, such as injuries or temporary soreness, defined as the numbers of participants who experienced an adverse event.
2. Self-reported satisfaction with the intervention, measured, e.g., with a Likert-type scale.

### Information sources and search strategy

#### Electronic searches

We will search the following electronic databases from their inception onwards: Cochrane Library (CENTRAL), MEDLINE via PubMed, Web of Science, CINAHL, PsycINFO, PEDro, SPORTDiscus, and Academic Search Premier. A sensitive search strategy was developed, using search terms (both MeSH terms and relevant keywords) related to the population/health problem of interest (back pain) and intervention setting (workplace). An example for the PubMed/MEDLINE search strategy can be viewed in Additional file 2.

We will conduct all searches without applying any language restrictions. We will, however, restrict the inclusion of studies to reports published in English or German. We will document any potentially relevant studies published in any other language for which an English title or abstract is available that allows for a preliminary judgement. We will exclude studies for which only a conference abstract or poster is available.

#### Searching other resources

We will check the reference lists of all eligible studies and of relevant existing systematic reviews for further relevant studies. Furthermore, we will conduct searches of the following sources to identify ongoing or yet unpublished studies: The International Clinical Trials Registry Platform of the World Health Organization (ICTRP), the German Clinical Trials Register (DRKS), and ClinicalTrials.gov. Additionally, we plan to contact experts in the field to enquire on the availability of further relevant studies.

### Data collection and analysis

#### Selection of studies

The selection process will be conducted in compliance with international standards [26] and using appropriate software (e.g., Endnote for the management of references and Covidence [27] for the screening process). Two review authors will independently assess potentially eligible trials for inclusion through a two-stage approach (screening of titles/abstracts followed by screening of potentially relevant full texts). Any disagreements at each stage will be resolved through discussion and involving a third review author, where needed.

#### Data extraction and management

Two review authors will independently extract data using a purpose-developed and piloted data extraction sheet. We plan to extract the following key details for each included study: first author and publication year (study ID), study design, country, study duration (from the first enrollment of participants to the last follow-up), sample sizes in intervention groups, participants’ age, gender and highest level of education, type of work, work setting, description of interventions and comparisons, outcomes (including information on measurement instruments, assessors and timing of measurement), and results for each outcome of interest and each study group (for continuous outcomes: mean values with standard deviations (SDs) or standard errors (SEs), mean differences (MDs) with 95% confidence intervals (CIs) or *p* values; for dichotomous outcomes: numbers and percentages of outcome events, risk ratios (RRs) with 95% CIs, or *p* values). We will further extract data on funding sources and potential conflicts of interest for each included study. Any discrepancies will be resolved through discussion, or, if needed, by involving a third review author. In case of multiple reports for a single study, we will aggregate the available information.

If repeated measurements of the same outcomes are reported, we will extract outcome data for all time points available. We expect high variability in the length of follow-ups between the original studies. As combining outcomes assessed at different time points might be not informative [24], we plan to divide the follow-up measurements into appropriate categories, i.e., short term (< 6 months from baseline), medium term (6 to < 12 months from baseline), and long term (≥ 1 year from baseline) [28]. We plan to conduct our main analysis using the long-term outcomes. The remaining follow-up categories will be considered in sensitivity analyses. Depending on the available measurement points in the original studies, we may decide to choose a different follow-up category for our main analysis in order to include more data. This decision will be made before any meta-analyses are conducted.

#### Assessment of risk of bias in included studies

Each included study will be independently assessed for risk of bias (RoB) by two review authors using the revised Cochrane tool for assessing RoB in randomized trials, RoB 2 [29]. The RoB assessment will be conducted and documented separately for each outcome of interest. The RoB 2 tool comprises five RoB domains: (1) bias arising from the randomization process, (2) bias due to deviations from intended interventions, (3) bias due to missing outcome data, (4) bias in measurement of the outcome, and (5) bias in selection of the reported result [29]. The RoB 2 assessment of cluster RCTs includes an additional domain: bias arising from identification or recruitment of individual participants within clusters [29]. RoB is rated for each domain, and the ratings for all domains are then used for an overall RoB judgement, which may be “low RoB”, “some concerns” or “high RoB” [29]. Any discrepancies in ratings between the review authors will be discussed and resolved, if necessary, involving a third review author.

#### Measures of treatment effect

We will use RRs with 95% CIs for dichotomous data and MDs with SDs for continuous data. In cases where an outcome has been obtained using different measurements (e.g., pain measured using different pain scales), we will use Hedges’ *g* as standardized mean difference (SMD). Where available, we will give preference to mean change scores from baseline to follow-up over mean scores at follow-up.

#### Unit of analysis issues

For cluster RCTs, we will check whether study authors applied an appropriate analysis method to account for clustering, such as an analysis of covariance that takes account of baseline cluster differences [30]. If this is not the case, we will adjust the data as described in the Cochrane Handbook [31].

#### Dealing with missing data

We plan to contact study authors in case of missing or unclear data. In cases where SEs are reported instead of SDs, we will convert them to SDs [32]. In cases where neither SDs nor SEs are available, we will calculate SDs using reported CIs or *p* values as described in the Cochrane Handbook [33]. In cases where none of the procedures described above are possible, we will estimate missing SDs using a validated imputation technique based on the reported SDs of the other included studies [34].

#### Assessment of risk of publication bias

To assess risk of publication bias, we plan to examine funnel plots for each pairwise meta-analysis and comparison-adjusted funnel plots for each NMA [35]. Additionally, we plan to conduct Egger’s linear regression test for funnel plot asymmetry [36]. We will only assess publication bias for outcomes for which at least 10 studies are available [37].

#### Data synthesis

In pairwise meta-analyses, the effects of an intervention versus a control condition are directly compared [38]. Since work-related preventive interventions are diverse and often contain several intervention components [39], an alternative approach is needed that allows comparison of multiple intervention strategies. We therefore plan to perform a NMA to combine all available evidence from direct and indirect comparisons across a network of trials [16].

We plan to conduct three steps of analysis:

1. First, we will conduct pairwise meta-analyses for all direct comparisons of interventions with other interventions or comparators that have been investigated in at least two original studies. In cases where study effects vary considerably in terms of the direction and/or size, we may, though, decide against meta-analysis and for a descriptive analysis. Based on the expectation that the intervention effects of the included studies are likely to vary to some extent, we plan to conduct the meta-analyses using a random-effects model. The ultimate decision about the most appropriate approach for each analysis, though, will be made based on the characteristics of the available studies and their observed effects, i.e., in particular based on the number of available studies for the meta-analyses and the extent of variation in the direction and size of the observed effects. Separate meta-analyses will be conducted for each pre-specified outcome. We will present the results of each pairwise meta-analysis using forest plots. Statistical heterogeneity among the studies will be investigated using Cochran’s *Q* test and the *I*^2^ statistic [40]. For the interpretation of the *I*^2^ statistic, we will follow the guidance provided in the Cochrane Handbook [41]. If we find considerable statistical heterogeneity for a direct comparison, we will not perform meta-analysis and instead conduct a descriptive analysis for the respective comparison.
2. In a next step, we plan to conduct NMAs using a frequentist approach [42]. We will include all available interventions characterized according to their content (as described above). Comparisons for which no meta-analysis will be performed (due to the reasons mentioned above) will be excluded from the NMAs. The network structure for each outcome will be illustrated using network graphs [38]. The size of each node in the network graph will correspond to the total number of participants allocated to the respective intervention and the width of each line will correspond to the number of studies contributing to the respective direct comparison [38]. Network graphs with colored edges according to quality items will be generated to evaluate potential biases [35]. Quality items will include the domains of RoB 2 [29] described above. In a league table, we will provide the summary effect size along with its 95% CI for each available comparison of interventions [16]. For each NMA, we will calculate *P* scores for interventions, which are a frequentist version of the surface under the cumulative ranking curve (SUCRA) [42]. *P* scores may take values between 0 and 1, with 0 indicating that an intervention is “always worst” and 1 indicating that an intervention is “always best” compared to the other interventions considered [38]. Interventions will be ranked according to their *P* scores for each outcome considered.
3. To evaluate the effects of single components in multicomponent interventions, we plan to perform an additional CNMA [17]. In classical NMA, combined interventions such as exercise program plus education are considered as independent interventions (i.e., exercise program plus education = intervention AB) [17]. CNMAs break down interventions into their individual components (e.g., exercise program = intervention component A; education = intervention component B) [17]. The additive CNMA model assumes that the effect of a combined intervention AB is the sum of the effects of the individual components A and B [17]. We will apply the additive model to investigate the relative effects of intervention components of the included studies.

All analyses will be performed with the statistical software R using the R packages meta [43] and netmeta [44].

#### Subgroup analysis

To investigate possible differences in the effects of interventions for specific subgroups, we plan to perform NMAs for the following subgroups:

- Job exposure: we plan to determine job exposure in a two-step procedure: first, we will classify the occupations of the study populations using the International Standard Classification of Occupations (ISCO-08) [45]. Participants’ overall job exposure (including physical and psychosocial exposure) will then be rated using the Job Exposure Matrices (JEM) for ISCO by Kroll [46] resulting in ratings of either low, medium, or high. The JEM have been shown to be a valid instrument to classify work demands in the context of health science [47].
- Intervention duration: for this subgroup-analysis, we plan to categorize interventions according to their total duration, e.g., < 1 month, 1–3 months, > 3 months.
- Baseline back pain: we plan to differentiate between samples without back pain at baseline and mixed samples including both, people with and without back pain at baseline.
- Localization of back pain: for this subgroup-analysis, we will subdivide studies according to the reported localizations of back pain, measured as an outcome of the intervention (e.g., low back pain and neck pain).
- Gender: this subgroup-analysis will include all studies that exclusively investigated a specific gender or reported gender-specific subgroup-analyses.

Furthermore, we plan to conduct NMA regressions for mean age and the proportion of female participants to explore the impact of these possible moderator variables using WinBUGS [48].

#### Sensitivity analysis

To assess the impact of RoB on our results, we plan to conduct sensitivity analyses in which we will exclude studies with high RoB in at least one domain from the NMAs. To account for a possible impact of follow-up duration, we plan to conduct additional NMAs for the remaining follow-up categories described beforehand, i.e. short term and medium term.

#### Assessment of transitivity

The assumption of transitivity, also referred to as “similarity”, implies that studies comparing different sets of interventions are sufficiently similar to allow indirect comparisons (i.e., comparing two interventions via a third one) [16, 38]. To detect potential intransitivity, we will assess the distribution of possible effect modifiers across all direct comparisons prior to conducting NMA [24], e.g., age, gender, duration of interventions, mode of delivery of interventions (e.g., supervised or self-directed), and study setting. If distributions are comparable across the available direct comparisons, we will assume that the assumption of transitivity is met.

#### Assessment of consistency

Consistency indicates that results of direct and indirect comparisons are in agreement so that they can reasonably be combined in a NMA [49]. We will evaluate consistency using the node splitting approach that separates comparisons into direct and indirect information from each node [50]. Furthermore, we will consider a design-by-treatment interaction model to test for design inconsistency in the whole network [49]. Any inconsistency will be explored using subgroup analyses or meta-regressions as described before. In case of substantial unexplained inconsistency, we will not report results of the respective NMA.

#### Rating the certainty of the evidence

We will apply the GRADE (Grading of Recommendations Assessment, Development and Evaluation) approach to rate the certainty of the evidence derived from our NMAs [51, 52]. Following the approach for rating NMA results described by the GRADE working group [51], the certainty of direct effect estimates will be rated in a first step, considering RoB, inconsistency, indirectness, and publication bias. These ratings will inform the rating of the indirect estimates; additionally, intransitivity will be considered for indirect evidence [52]. Based on the ratings of direct and indirect evidence, the certainty of the network effect estimates will be rated [51, 52]. In case of considerable imprecision or incoherence, we will rate down certainty of the network estimates [51, 52]. Applying the GRADE approach results in four possible levels of certainty: high, moderate, low, and very low [51, 52]. The rating will be conducted independently by two review authors for each outcome considered. Any disagreement will be discussed and resolved by consensus, if necessary with a third review author. If no NMA can be performed, we will only rate the certainty of the evidence of our pairwise meta-analyses.

## Discussion

This planned systematic review and NMA aims to identify effective work-related interventions for preventing non-specific back pain. We plan to synthesize the currently available evidence to inform guidelines as well as relevant stakeholders including those directly affected and political and economic decision-makers. By using NMA and CNMA, we expect to gain novel and important insights that go beyond the available systematic reviews [9, 11, 19]. Ranking interventions according to their relative effects [16] may enable stakeholders to select the most promising interventions and to foster a more targeted use of resources. Using CNMA, we plan to break down multicomponent interventions into their individual components and determine their relative effects [17]. We expect that the identification of effective intervention components within the available interventions may particularly inform the further development and implementation of effective (multicomponent) work-related interventions for preventing back pain as well as future research in this field. Subgroup analyses to determine whether and how intervention effects differ between groups with different job exposure may enable an optimal adaption of future interventions to the specific needs of different working populations or settings. Transferring our results into practice, they may, in the long term, lead to an improvement in health and potentially a reduction in medical expenses.

Some potential limitations at individual study level as well as at review level can be anticipated and should be noted. Limitations at individual study level may relate to reporting issues, such as poor reporting of study characteristics or a lack of data in a form that we can use for our analyses. However, in case of missing outcome data, we will contact study authors to request the required data. Whether studies can be included in our subgroup analyses will also depend on their reporting of details such as participant and intervention characteristics. Deciding whether to group interventions together or split them into different nodes can be challenging when conducting NMA [24]. We expect to include a range of different interventions that may be heterogeneous in both content and delivery. We have presented the planned clusters of interventions in the methods section. However, depending on our findings, it may be necessary to make further decisions, e.g., regarding to the question whether to include further intervention categories or consider different modes of delivery. Our rationales and final decisions on this will be presented in the report of this systematic review. To create optimal transparency regarding the conduct of this systematic review, we plan to publish an example of our extracted data as well as our analytical code as online supplementary material upon completion and publication of this systematic review.

Overall, we are confident that our results will contribute to improving work-related prevention strategies and advancing future research in terms of content and methodology.

## Supplementary Information

**Additional file 1.** PRISMA-P 2015 Checklist

**Additional file 2.** Search strategy for the electronic database PubMed/MEDLINE.

## Data Availability

Not applicable.

## Abbreviations

CI: Confidence interval
GRADE: Grading of Recommendations Assessment, Development and Evaluation
NMA: Network meta-analysis
CNMA: Component network meta-analysis
ISCO: International Standard Classification of Occupations
JEM: Job exposure matrices
MD: Mean difference
RCT: Randomized controlled trial
RoB: Risk of bias
RR: Risk ratio
SD: Standard deviation
SE: Standard error
SMD: Standardized mean difference
SUCRA: Surface under the cumulative ranking curve
