# Supplementary material for: Work-related interventions for preventing back pain—protocol for a systematic review and network meta-analysis
Source: Syst Rev. 2021 Aug 30;10:241. doi: 10.1186/s13643-021-01768-5 (PMC8406587; doi:10.1186/s13643-021-01768-5)
Supplement: Supplementary file 2 — Additional file 2. Search strategy for the electronic database PubMed/MEDLINE. [file 13643_2021_1768_MOESM2_ESM.docx]

**Additional file 2**

Search strategy for the electronic database PubMed/MEDLINE.

| **Search** | **Query** |
| --- | --- |
| #15 | Search: **#13 AND #14** Sort by: **Most Recent** |
| #14 | Search: **randomized controlled trial[Publication Type] OR random allocation [MeSH Terms] OR controlled clinical trial[Title/Abstract] OR random*[Title/Abstract]** Sort by: **Most Recent** |
| #13 | Search: **#9 OR #12** Sort by: **Most Recent** |
| #12 | Search: **(#1 OR #10) AND #11** Sort by: **Most Recent** |
| #11 | Search: **(prevent*[Title/Abstract] OR prophyla*[Title/Abstract])** Sort by: **Most Recent** |
| #10 | Search: **„Back pain"[Title/Abstract] OR „ low back pain"[Title/Abstract] OR „back ache*"[Title/Abstract] OR back dysfunction*[Title/Abstract] OR back pain*[Title/Abstract] OR back strain*[Title/Abstract] OR backache*[Title/Abstract] OR low back ache*[Title/Abstract] OR low back syndrome*[Title/Abstract] OR low backpain*[Title/Abstract] OR lowback pain*[Title/Abstract] OR lower back pain*[Title/Abstract] OR lower backache*[Title/Abstract] OR lower backpain*[Title/Abstract] OR lumbago*[Title/Abstract] OR lumbal pain*[Title/Abstract] OR lumbal syndrome*[Title/Abstract] OR lumbalgia*[Title/Abstract] OR lumbar pain*[Title/Abstract] OR lumbar spine syndrome*[Title/Abstract] OR lumbar syndrome*[Title/Abstract] OR lumbodynia*[Title/Abstract] OR lumbosacral pain[Title/Abstract] OR musculoskeletal pain[Title/Abstract]** Sort by: **Most Recent** |
| #9 | Search: **#5 AND #8** Sort by: **Most Recent** |
| #8 | Search: **#6 OR #7** Sort by: **Most Recent** |
| #7 | Search: **Workplace[Title/Abstract] OR work setting[Title/Abstract] OR work site[Title/Abstract] OR work environment[Title/Abstract] OR industry[Title/Abstract] OR company[Title/Abstract] OR factory[Title/Abstract] OR office[Title/Abstract] OR offices[Title/Abstract] OR computer user*[Title/Abstract] OR laborer[Title/Abstract] OR employ*[Title/Abstract] OR personnel [Title/Abstract] OR occupation*[Title/Abstract] OR job [Title/Abstract] OR jobs[Title/Abstract] OR profession*[Title/Abstract] OR staff[Title/Abstract]** Sort by: **Most Recent** |
| #6 | Search: **Workplace[MeSH Terms] OR Occupational Health[MeSH Terms] OR Occupational Diseases / prevention & control*** Sort by: **Most Recent** |
| #5 | Search: **#1 OR #4** Sort by: **Most Recent** |
| #4 | Search: **#2 AND #3** Sort by: **Most Recent** |
| #3 | Search: **pain[Title/Abstract] OR discomfort[Title/Abstract] OR ache[Title/Abstract] OR sore*[Title/Abstract] OR injur*[Title/Abstract] OR symptom* [tiab] OR disorder* [tiab] OR problem* [tiab]** Sort by: **Most Recent** |
| #2 | Search: **back[Title/Abstract] OR lumbar[Title/Abstract] OR neck[Title/Abstract] OR cervical[Title/Abstract] OR musculoskeletal[Title/Abstract]** Sort by: **Most Recent** |
| #1 | Search: **Low Back Pain[MeSH Terms] OR Back Pain[MeSH Terms] OR Neck Pain[MeSH Terms] OR Musculoskeletal Pain[MeSH Terms]** Sort by: **Most Recent** |
